# Alfalfa Xeno-miR168b Target *CPT1A* to Regulate Milk Fat Synthesis in Bovine Mammary Epithelial Cells

**DOI:** 10.3390/metabo13010076

**Published:** 2023-01-03

**Authors:** Jingying Jia, Hongjuan Duan, Baobao Liu, Yanfen Ma, Yun Ma, Xiaoyan Cai

**Affiliations:** 1 School of Agriculture, Ningxia University, Yinchuan 750021, China; 2 Key Laboratory of Ruminant Molecular Cell Breeding of Ningxia Hui Autonomous Region, Yinchuan 750021, China

**Keywords:** milk fat, alfalfa, xeno-miR168b, cross-kingdom, *CPT1A*

## Abstract

It was shown that microRNAs (miRNAs) play an important role in the synthesis of milk fat; thus, this manuscript evaluated whether exogenous miRNA (xeno-miRNAs) from alfalfa could influence the milk fat content in dairy cows. At first, mtr-miR168b was screened from dairy cow milk and blood. Then, EdU staining, flow cytometry, Oil Red O staining, qRT-PCR, and WB were applied to explore the effect of xeno-miR168b on the proliferation, apoptosis, and lipid metabolism of bovine mammary epithelial cells (BMECs). Finally, in order to clarify the pathway that regulated the lipid metabolism of BMECs using xeno-miR168b, a double-luciferase reporter assay was used to verify the target gene related to milk fat. These results showed that overexpression of xeno-miR168b inhibited cell proliferation but promoted apoptosis, which also decreased the expression of several lipid metabolism genes, including *PPARγ*, *SCD1*, *C/EBPβ*, and *SREBP1*, significantly inhibited lipid droplet formation, and reduced triglyceride content in BMECs. Furthermore, the targeting relationship between *CPT1A* and xeno-miR168b was determined and it was confirmed that *CPT1A* silencing reduced the expression of lipid metabolism genes and inhibited fat accumulation in BMECs. These findings identified xeno-miR168b from alfalfa as a cross-kingdom regulatory element that could influence milk fat content in dairy cows by modulating *CPT1A* expression.

## 1. Introduction

MicroRNAs (miRNAs) are short, single-stranded non-coding RNAs that regulate the expression of up to 60% of the protein-coding genes in genomes [1]. Recent studies demonstrated that an animal’s diet might affect miRNA expression in milk, and exogenous miRNAs (xeno-miRNAs) from plants can also regulate gene expression in animals, which is a phenomenon known as ‘cross-kingdom’ gene regulation [2]. There are several classic examples of cross-kingdom regulation by miRNAs. For example, miR159a from soybean can inhibit NF-κB and TGF-β1 signaling in mice, which helps prevent hepatic fibrosis [3,4]. Another notable example is the ability of miR2911 in the decoction of honeysuckle to inhibit the replication of the influenza A virus, and it could also inhibit SARS-CoV-2 replication and accelerate the negative conversion of infected patients [5,6].

Several studies showed that plant-derived miRNAs can specifically regulate fat metabolism in animals. First, miR168 from rice can target the low-density lipoprotein (*LDL*) receptor adaptor protein (*LDLRAP1*) in the liver of mice and humans, which slows the clearance of blood *LDL*s [7]. Second, plant miR167e-5p can target β-catenin to promote fat production in 3T3-L1 cells [8]. Third, plant-derived miRNAs can significantly increase muscle lipid metabolism and reduce epididymal fat deposition in mice [9]. Fourth, plant-derived miRNAs can be detected in abundance in the adipose tissue of pigs fed with maize [10].

Based on strong evidence that plant-derived miRNAs can regulate lipid metabolism in animals, it is possible that plant-derived miRNAs can affect fat metabolism in bovine mammary epithelial cells (BMECs), and thus, regulate milk fat content. Indeed, multiple studies documented the presence of plant miRNAs in the milk of dairy cows, as well as the effect of miRNAs on milk quality [11,12,13]. Fat is the most variable component of milk, and it is influenced by many factors, including nutrition, genetics, physiological status, and environment. Among these, nutritional factors are the most influential [14,15,16]. During diet-induced milk fat depression, milk fat production is drastically reduced within a few days, in some cases becoming more than 50% depleted [17,18]. Therefore, understanding the molecular mechanisms by which diet can influence milk fat synthesis is essential for optimizing the nutritional content of milk from ruminant animals, as well as improving the fat content of dairy products.

Milk fat is the main nutritional component of milk and an important indicator of milk quality. In animal husbandry, alfalfa is considered highly nutritious and improves the nutrient content of the meat, eggs, and milk produced. When included in a balanced diet for dairy cows, increasing the amount of hay in the feed could proportionally improve milk quality; however, whether miRNAs from alfalfa can affect the milk quality through cross-kingdom regulation is not known [19,20,21,22]. Therefore, we set out to study the regulatory effect of alfalfa miRNAs on milk fat.

## 2. Materials and Methods

### 2.1. Alfalfa Extraction from Alfalfa

Alfalfa *cv.Zhongmu 1* (ZM1) and *cv.Xinyan 52* (XY52) strains were used for all the experiments in this study. Fresh whole alfalfa plants were collected from Ningxia Modern Science and Technology Park, China. The plants were immediately frozen in liquid nitrogen before use, transported, and stored at −80 °C in the laboratory. Three samples were processed for each strain (5 g each). Total RNA samples, including miRNAs, were extracted using Trizol. The RNA quality control was performed using agarose gel electrophoresis before the RNA sequencing analysis with Biomarker technologies (Beijing, China) using the Illumina platform. Low-quality reads, reads with unknown base contents of 10% or more, and reads without a 3′ joint sequence were removed. The 3′ joint sequence was then removed; then, all sequences shorter than 18 nucleotides (nt) or longer than 30 nt were removed. The remaining reads were considered high-quality clean reads.

### 2.2. Blood and Milk Collection from Holstein Cows

Milk and blood of Holstein cows from Helanshan Dairy Industry Co., Ltd., in Ningxia Nongken, China, were collected and analyzed. All samples were taken from the unified dairy farm. Dairy cows with similar parity, calving date, calving interval, and lactation days were selected and were evaluated to produce either high-fat milk or low-fat milk (Table 1). Samples were collected in December 2021. Blood was collected from the tail vein into a vacuum blood collection tube containing anticoagulant and stored at −20 °C. According to the milking schedule of the dairy factory, the morning, midday, and evening milk from the same dairy cow was collected and mixed according to the dairy herd improvement (DHI) testing and mixing standard of 4:3:3. The emulsion was stored at −80 °C until use. The experimental cows were fed the same balanced total mixed diet (Table 2).

### 2.3. Screening of Dairy Cows and qRT-PCR of Exogenous Xeno-miRNAs

The mixed milk emulsion was entrusted to Ningxia Agriculture and Rural Office for the DHI analysis. The specific analytical indicators included milk fat, milk protein, lactose, and total solid content. Three cows with a high milk fat percentage and three cows with a low milk fat percentage were selected for subsequent experiments.

Five alfalfa miRNAs were prioritized for this study: mtr-novel-miR54, mtr-miR156f, mtr-miR166a, mtr-miR168b, and mtr-miR168c-3p. The miRNAs were detected using Trizol and quantified via qRT-PCR using primers designed with Primer 5.0 and synthesized by Anhui General Biol (Table 3).

### 2.4. Oxidation Experiment

A total of 10 mM sodium periodate was added to 10 μL of extracted bovine blood RNA sample, mixed, and oxidized at −20 °C for 40 min. After centrifuging, 1 mL of absolute ethanol was added to resuspend the precipitate, which was incubated at −20 °C overnight and the RNA samples were centrifuged again at a low temperature. After absolute ethanol was discarded, 20 μL of enzyme-free water was added to dissolve the RNA precipitate, and the RNA in the samples was completely oxidized. The alfalfa-derived xeno-miR168b and bovine-derived bta-miR-16a were quantified in the oxidized RNA solution and the plant-derived miRNAs were verified (Table 3).

### 2.5. Cell Culture and Transfection

HEK-293T cell line and BMECs, which were used in the manuscript, were obtained from Ningxia University, Key Laboratory of Ruminant Molecular Cell Breeding of Ningxia Hui Autonomous Region. HEK-293T cells were cultured in DMEM high glucose (Hyclone, LA, USA) and 5% fetal bovine serum (FBS) (BI, Jerusalem, Israel) at 37 °C with 5% CO_2_ and saturated humidity. The BMECs were identified and frozen using liquid nitrogen [23]. The BMECs were cultured in 10% FBS at 37 °C with 5% CO_2_ and saturated humidity. To induce lactation, 5 μg/mL insulin (MCE, New Jersy, USA), 5 μg/mL hydrocortisone (MCE, New Jersy, USA), and 20 μg/mL prolactin (Prospec, Jerusalem, Israel) were added to a basic medium. Then, 48 h after transfection, an induction medium was added; this was recorded as day 0 of the induction. During the induction period, the medium was changed every other day. 

The sequence for the xeno-miR168b mimics was TCGCTTGGTGCAGGTCGGGAA. The sequences for si*CPT1A* were sense (5′-3′): GGGAGGAAAUCAAACCGAUTT and antisense (5′-3′): AUCGGUUUGAUUUCCUCCCTT. miRNA mimics and a miRNA negative control (NC) were purchased from Guangzhou RiboBio Co., Ltd. (RiboBio, Guangzhou, China), and si*CPT1A* and si-NC were purchased from Shanghai Sangon Biotech Co., Ltd. (Sangon, Shanghai, China). Cells were transfected with 50 nM xeno-miR168b mimics, 50 nM NC, 80 nM si*CPT1A*, or 80 nM si-NC using a lipofectamine 3000 (Invitrogen, CA, USA) transfection reagent for 48 h at 37 °C. Cells were used for studies after 48 h of transfection. The cells in the blank control group did not receive any treatment.

### 2.6. 5-Ethynyl-2′-Deoxyuridine (EdU) Staining

The proliferation of the BMECs was investigated using the BeyoClickTM EdU-555 Cell Proliferation Kit (Beyotime, Shanghai, China) according to the manufacturer’s protocol. The mtr-miR168b mimics and NC were transfected into the BMECs; after 48 h, the BMECs were washed twice with PBS. EdU working solution (10 μM) was added to the cells prior to incubation in the dark for 2 h. After 2 h, the prepared Click reaction solution was added and then cells were incubated in the dark for 30 min for cytoplasmic staining. After 30 min, the Click reaction solution was discarded, and then nuclear staining was performed using 1X Hoechst 33342. Treated cells were observed and photographed with a fluorescence microscope (Olympus Corporation, Tokyo, Japan). The cytoplasms of newly proliferated cells were identified using red fluorescence, and the nuclei of all cells were identified using blue fluorescence.

### 2.7. Cell Apoptosis and Cell Cycle Analysis

Cell apoptosis and cell cycle phase were detected via flow cytometry using the Annexin V-FITC apoptosis detection kit (C1062M, Beyotime, China) and the cell cycle and apoptosis analysis kit (C1052, Beyotime, China), respectively, according to the manufacturer’s protocols. To detect apoptosis, cells were digested with trypsin and then a culture medium was added to stop digestion. Cells were collected in 1.5 mL centrifuge tubes spun at 1000 g for 5 min, the supernatant was removed, and the pellet was resuspended in 1 mL osis. Cells were collected via centrifugation and discarding the supernatant as before, and 195 μL Annexin V-FITC binding solution, 5 μL Annexin V-FITC, and 10 μL propidium iodide (PI) were added to the cell pellet. After incubation at room temperature for 20 min in the dark, cells were analyzed using flow cytometry. 

The determination of the cell cycle stage was consistent with the early stage of cell apoptosis. To determine the cell cycle phase, the cells were centrifuged again and the PBS was discarded after cell suspension; then, they were put into a water bath. Then, 70% ethanol was added and the solution was blown and mixed and then fixed at 4 °C for 24 h. After fixation, the cells were centrifuged again, ethanol was sucked and discarded, and PBS was added to resuspend and wash the cells and then discarded. After 500 μL propidium iodide staining solution was added to each tube cell, the cell cycle was determined via flow cytometry.

### 2.8. Gene Expression Assay

Total RNA from the BMECs transfected with xeno-miR168b mimics and NC was extracted using Trizol, reverse-transcribed into cDNA, and then the gene expression levels in different treatment cells were quantitatively detected. Detection genes indicating proliferation, cell cycle, apoptosis, and associated with lipid metabolism were quantified via qRT-PCR. The primer sequences used in the study are shown in Table 4. The expression level of lipid-metabolism-related genes in BMECs transfected with si-NC and siCPT1A were also quantitatively detected.

### 2.9. Western Blot Assay

The BMECs were collected in 0.25% trypsin (Solarbio, Beijing, China) and Western blot analysis was performed using standard techniques reported by Hou [24]. The protein concentration was determined using a BCA protein determination kit (KeyGEN BioTECH Bio, Jiangsu, China). A total of 100 μg protein extracts were loaded, then separated using 10% SDS polyacrylamide gel electrophoresis and transferred onto polyvinylidene difluoride (PVDF) membranes (Epizyme Bio, Shanghai, China). The extracts were blocked in a blocking solution at room temperature for 15 min and the membranes were incubated with antibodies. Primary antibodies against *GAPDH* (AB0036,1:3000, Abways, Shanghai, China), *PCNA* (D220014, 1:500, Sangon Biotech, Shanghai, China), *BAX* (CY5059, 1:500, Abways, Shanghai, China), *AMPK* (AP0569, 1:500, Bioworld, Shanghai, China), and *p-AMPK* (BS4010, 1:500, Bioworld, Shanghai, China) were used, and the secondary antibody was goat anti-rabbit IgG (ZB-2301, 1:20000, ZSGB-Bio, Beijing, China). A chemiluminescent ECL Western blot system (Tanon-5200, Shanghai, China) was used for signal detection, and protein abundance was measured using ImageJ software.

### 2.10. Oil Red O Staining and Triglyceride Assay

Lipid droplets were detected using Oil Red O stain kit (G1262, Solarbio, Beijing, China) according to the manufacturer’s protocols. Cells induced for 0 d, 4 d, and 8 d were washed with PBS and fixed in 4% paraformaldehyde for 15 min. After washing again with PBS, Oil Red O staining solution was added and the cells were incubated for 15 min. The cells were then washed with PBS until no excess staining solution was detected, and then Mayer Hematoxylin staining solution was added to stain the nucleus. The cells were again washed in PBS to remove the Mayer Hematoxylin staining solution and the cell lipid content was observed under an inverted fluorescence microscope (Olympus Corporation, Tokyo, Japan).

Cellular triglycerides (TGs) were detected in the BMECs with a high expression of xeno-miR168b. The cells were collected and crushed using ultrasonic waves (intensity 20%, ultrasonic wave for 2 s, rest for 1 s, repeated for 1 min) and then centrifuged at 8000 *g* centrifugation for 10 min. The supernatant was collected and processed for TG detection using the triglycerides content detection kit (no. SH312K, G-CLONE, Beijng, China) according to the manufacturer’s instructions. Absorbance was measured in room-temperature samples at 420 nM and the TG content was calculated as follows:

TG content (mg/10^6^ cell) = (Absorbance-sample − Absorbance-blank)/(Absorbance- TG standard solution − Absorbance-blank)/cell density.

### 2.11. Dual-Luciferase Reporter Gene Assays

The target gene of xeno-miR168b was predicted using TargetScan database (https://www.targetscan.org/, accessed on 1 November 2022). The target gene CPT1A, which is involved in the related signal pathway of regulating milk fat synthesis, was screened out. Dual-luciferase reporter constructs were generated by cloning 3′-UTR wild-type (WT) and mutant-type (MUT) *CPT1A* sequences (ShengGong, China) into the Psi-checkⅡ vector (Promega, Madison, WI, USA) at the NotI and XhoI (Promega, Madison, WI, USA) restriction sites. HEK-293T cells were grown in 6-well plates and transfected at 80% confluence using a Lipofectamine 3000 reagent (Invitrogen, Waltham, MA, USA). The miRNA-sequence- and luciferase-containing vectors were co-transfected into HEK-293T cells and the gene expression and luciferase activity were detected after 48 h.

### 2.12. Statistical Analysis

At least three biological and three technical replicates were performed for each experiment, and the relative expression was calculated using the 2^−ΔΔcq^ formula. The test results were plotted and analyzed for significance using GraphPad Prism8 software, and *p* < 0.05 was considered statistically significant. Statistical significance between two groups was determined using Student’s *t*-test, and for more than two groups, it was determined using 2-way ANOVA.

## 3. Results and Analysis

### 3.1. Alfalfa miRNA Screening

**Figure 1:**
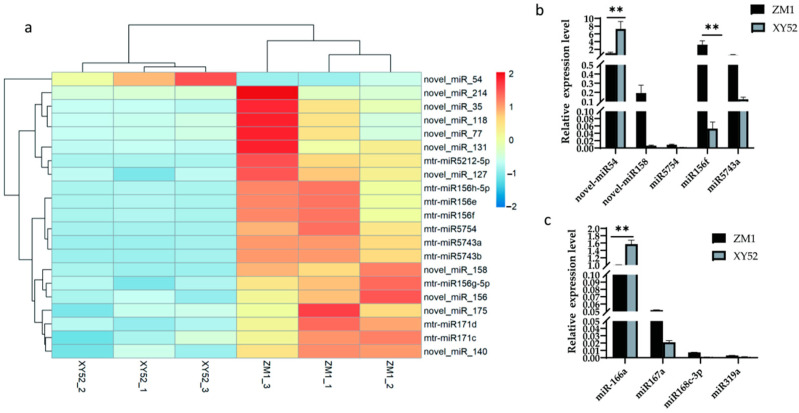
Differentially expressed miRNAs in alfalfa were determined using RNA-seq, and highly expressed miRNAs were quantified using RT-qPCR. Data are presented as the mean ± SD for *n* = 3/group. ** *p* < 0.01 was considered statistically significant. (**a**) A heat map of differentially expressed miRNAs in alfalfa, (**b**) relative expression levels of differentially expressed miRNAs, and (**c**) relative expression levels of cross-kingdom-regulated miRNAs.

We identified twenty-one miRNAs that were differentially expressed in ZM1 and XY52 alfalfa samples that may be the key to the milk quality difference between ZM1- and XY52-fed dairy cows (Figure 1a). Considering that the higher the TPM (transcripts per million) value of miRNAs in RNA-seq results, the higher the abundance of miRNAs and the greater the possibility of their retention after being ingested by dairy cows, five miRNAs with high TPM in ZM1 and differential expression in ZM1 and XY52 were selected, namely, novel-miR54, novel-miR158, miR5754, miR156f (miR156e, miR156h-5p), and miR5743a (miR5743b) (Table 5). We confirmed their expression levels via RT-qPCR, validating the RNA-seq results. As shown in Table 5, the expression level of novel-miR54 was significantly upregulated in XY52 compared with that in ZM1 (*p* < 0.01). While the expression of miR156f was significantly higher in ZM1 compared with that in XY52 (*p* < 0.01; Figure 1b) and was the highest among the five miRNAs. (*p* < 0.01; Figure 1b). In addition, miR166a [25], miR167a [11], miR168c-3p [11], and miR319a [26], which are known to have cross-kingdom regulatory effects, were quantitatively analyzed. The results showed that the expression of miR166a was the highest in the same variety, and it was significantly different between different cultivars (*p* < 0.01; Figure 1c).

Therefore, three miRNAs, namely, novel-miR54, miR156f, and miR166a, were prioritized for the subsequent experiments.

### 3.2. Xeno-miRNA Was Detected in Dairy Cow Milk and Blood 

**Figure 2:**
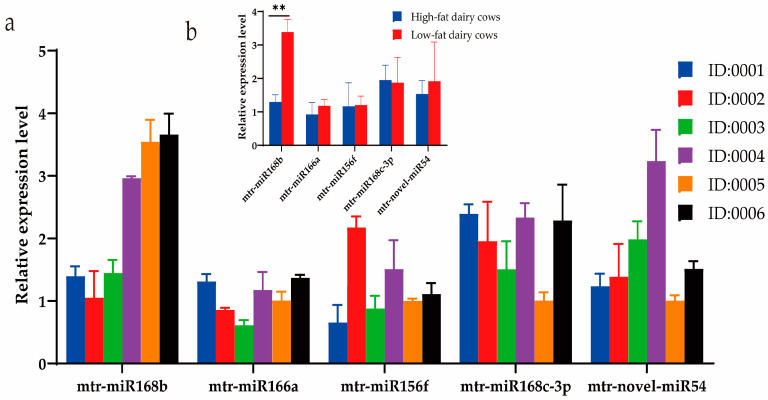
Relative expression of alfalfa miRNAs in bovine blood determined using qRT-PCR. Data are given as the mean ± SD for *n* = 3/group. ** *p* < 0.01 was considered statistically significant. (**a**) miRNA expression levels in blood and (**b**) significant difference in miRNA expression between high-fat-milk and low-fat-milk dairy cows.

**Figure 3:**
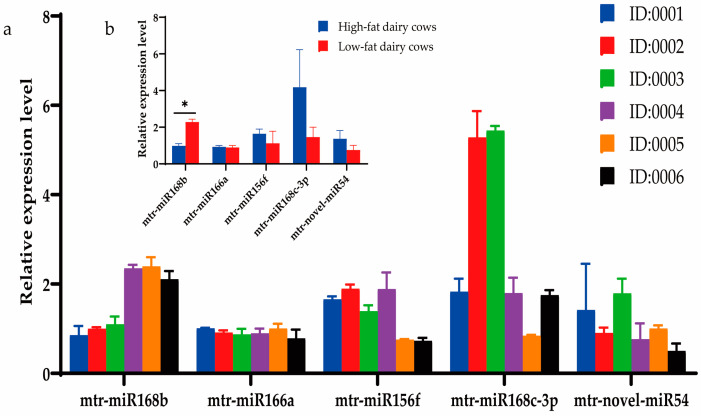
Relative expression of alfalfa miRNAs in milk as determined using qRT-PCR. Data are given as the mean ± SD for *n* = 3/group. * *p* < 0.05 was considered statistically significant. (**a**) miRNA expression levels in milk and (**b**) significant difference in miRNAs between high-fat-milk and low-fat-milk dairy cows.

**Figure 4:**
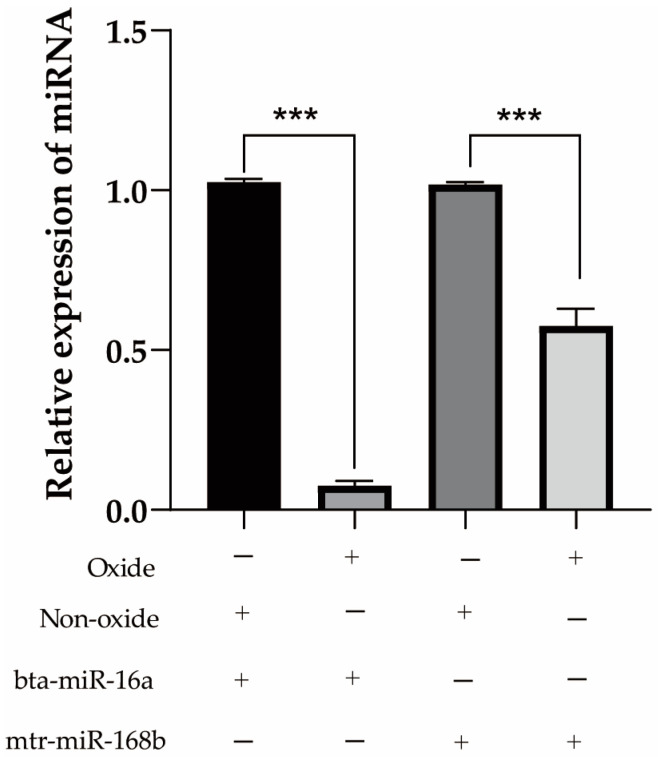
Detection of endogenous and exogenous miRNA expression levels in bovine blood before and after oxidation. Data are given as the mean ± SD for *n* = 3/group. *** *p* < 0.01 was considered statistically significant.

Milk from a cohort of dairy cows was analyzed using DHI to determine the fat content, and three cows each with high-fat (>4.2%; cows 0001, 0002, 0003) or low-fat (<3.6%; cows 0004, 0005, 0006) milk were selected for further study. The DHI test results of six cows are listed in Table 6. Expression of the three prioritized alfalfa miRNAs (mtr-novel-miR54, mtr-miR156f, mtr-miR166a), mtr-miR168b, and mtr-miR168c-3p, which belong to the miR168 family with cross-kingdom regulatory ability, were detected in the blood and milk of high-fat-milk and low-fat-milk dairy cows. The expression of mtr-miR168b was significantly lower in high-fat-milk dairy cows compared with low-fat-milk dairy cows in blood (*p <* 0.01; Figure 2) and in milk (*p <* 0.05; Figure 3). An oxidation test showed that compared with endogenous bta-miR-16a, plant-derived xeno-miR168b still had a higher expression level in oxidized bovine blood RNA (Figure 4), which showed that xeno-miR168b in bovine blood came from plants and could resist the oxidation of sodium periodate. Thus, alfalfa-derived xeno-miR168b was screened for subsequent test validation.

### 3.3. Xeno-miR168b Inhibited BMEC Proliferation but Promoted Apoptosis

**Figure 5:**
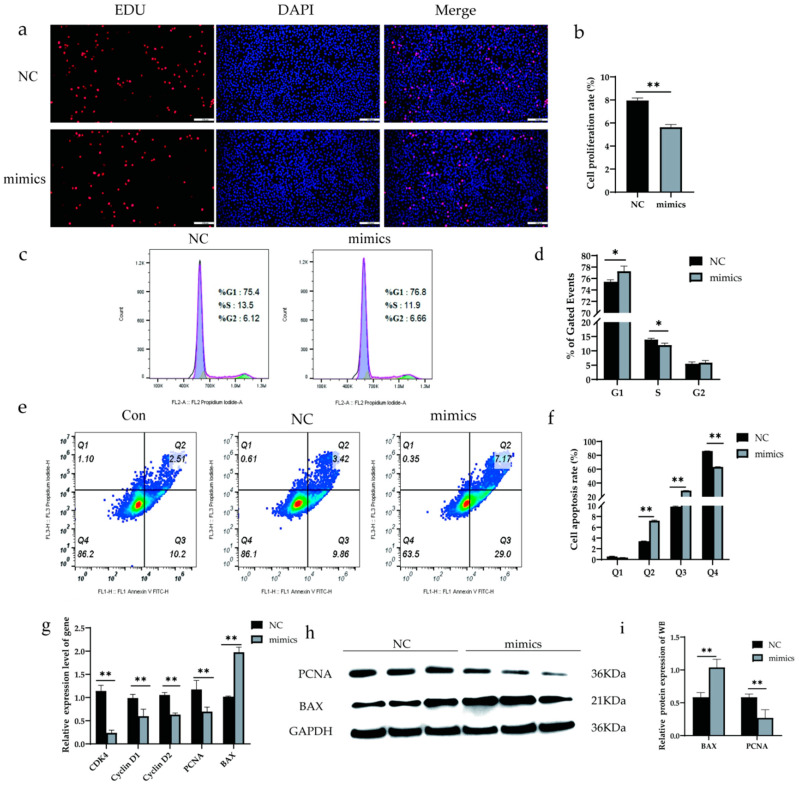
Overexpression of xeno-miR168b inhibited proliferation and promoted apoptosis in BMECs. Data are given as the mean ± SD for *n* = 3/group. * *p* < 0.05 or ** *p* < 0.01 were considered statistically significant. (**a**,**b**) Cell proliferation detected via EdU analysis; (**c**,**d**) flow cytometry analysis of cell cycle in BMCEs treated as indicated; (**e**) proportion of apoptotic cells detected via flow cytometry, where Q1 is necrotic cells, Q2 is early apoptotic cells, Q3 is late apoptotic cells, and Q4 is normal cells; (**f**) quantitative analysis of the cell apoptosis rate; (**g**) expression of apoptosis-related genes determined via qRT-PCR; (**h**) WB analysis of *PCNA* and *BAX*; and (**i**) quantitative analysis of protein bands in (**g**).

To determine whether alfalfa miRNAs may have biological effects in dairy cows, xeno-miR168b mimics and NC were transfected into BMECs, and then cell proliferation, cell cycle, and apoptosis were detected after 48 h. Xeno-miR168b significantly inhibited cell proliferation compared with NC (*p* < 0.05; Figure 5a,b). Furthermore, the number of cells in the S phase decreased and the number of cells in the G1 phase increased in xeno-miR168b-transfected cells compared with the controls, indicating that xeno-miR168b prolonged the G1 phase (Figure 5c,d). Xeno-miR168b also significantly increased the number of cells in early and late apoptosis compared with the controls (Figure 5e). 

We next analyzed the expression levels of genes related to cell proliferation (*CDK4*, *Cyclin D1*, *Cyclin D2*, *PCNA*) and apoptosis (*BAX*). Consistent with the phenotypes observed, the expression levels of *CDK4*, *Cyclin D1*, *Cyclin D2*, and *PCNA* were significantly decreased in xeno-miR168b-transfected BMECs compared with the controls (*p* < 0.01), whereas *BAX* expression was significantly increased in the xeno-miR168b-transfected BMECs compared with the controls (*p* < 0.01; Figure 5f). Western blot analysis confirmed the downregulated *PCNA* protein and upregulated *BAX* protein in xeno-miR168b-transfected BMECs compared with the control levels (Figure 5g,h). Together, our data suggested that xeno-miR168b could affect the biological process of BMECs by inhibiting proliferation, prolonging G1 the cell cycle, and inducing cell apoptosis. 

### 3.4. Xeno-miR168b Expression Regulated Genes Related to Lipid Metabolism in BMECs

**Figure 6:**
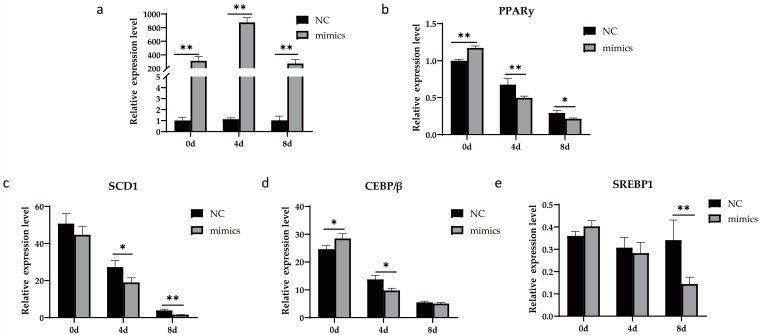
Overexpression of xeno-miR168b inhibited the expression of adipogenic marker genes in BMECs. Data are given as the mean ± SD for *n* = 3/group. * *p* < 0.05 or ** *p* < 0.01 were considered statistically significant. (**a**) Xeno-miR168b expression levels for 8 d after induction and (**b**–**e**) expression of genes related to lipid metabolism in mtr-miR168b-transfected or NC-transfected BMECs.

To determine whether the overexpression of xeno-miR168b can influence milk fat content from dairy cows, we analyzed changes in the expression levels of four genes related to milk fat metabolism in BMCEs transfected with xeno-miR168b and compared this to NC. Importantly, we used qRT-PCR to construct a longitudinal expression profile of the xeno-miR168b mimics in BMECs; we confirmed significant upregulation of the transcript as late as 8 d after induction (*p* < 0.05; Figure 6a). Xeno-miR168b decreased the expression of several lipid-metabolism-related genes, including *PPARγ*, *SCD1*, *CEBP/β*, and *SREBP1*, compared with the NC group (Figure 6).

### 3.5. Overexpression Xeno-miR168b Inhibited Lipid Droplet Formation and Reduced TG Content in BMECs 

**Figure 7:**
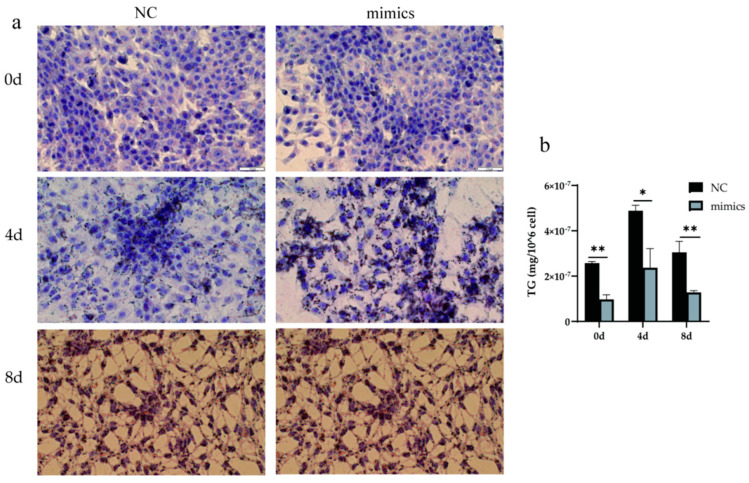
Overexpression of xeno-miR168b inhibited lipid droplet formation (**a**) and decreased TG content (**b**) in BMECs. Data are given as the mean ± SD for *n* = 3/group. * *p* < 0.05 or ** *p* < 0.01 was considered statistically significant.

To understand how alfalfa xeno-miR168b affects lipid metabolism in bovine cells, the experiment examined the lipid droplet accumulation and TG content over time in BMECs after the cells were transfected with xeno-miR168b mimics or NC control. At baseline (0 days of induction and transfected for 48 h), there were few lipid droplets and the droplets were small, both in the xeno-miR168b-transfected and NC-transfected BMECs. Four days after induction, significantly bigger lipid droplets were observed in the xeno-miR168b-transfected cells compared with the control group, but this difference resolved by 8 days after induction (Figure 7a). The TG content was significantly lower at all time points in the xeno-miR168b-transfected group compared with the NC group (*p* < 0.01; Figure 7b). Collectively, our findings demonstrated that the overexpression of xeno-miR168b inhibited fat synthesis and accumulation in BMECs.

### 3.6. Xeno-miR168b Targeting CPT1A

**Figure 8:**
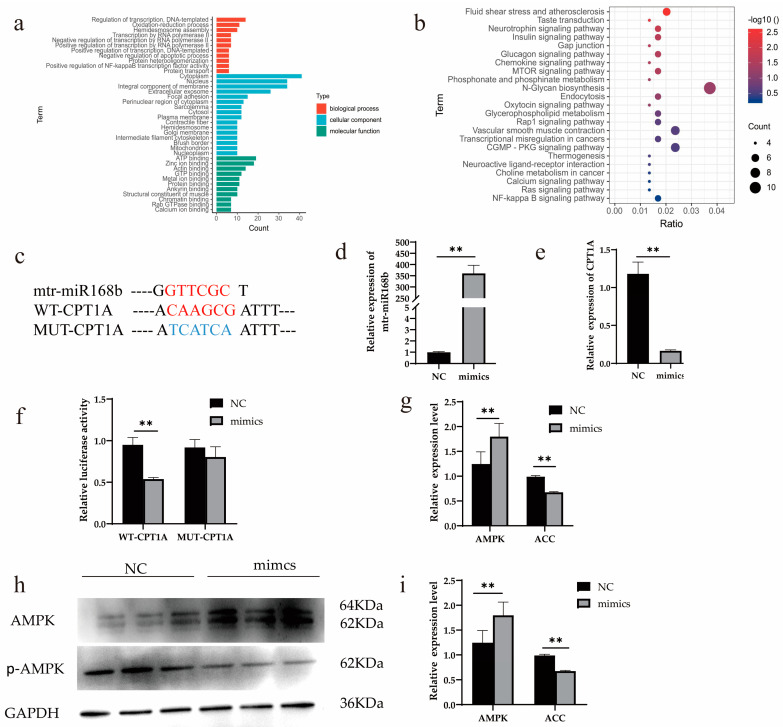
Xeno-miR168b regulated *CPT1A* expression. Data are given as the mean ± SD for *n* = 3/group. ** *p* < 0.01 was considered statistically significant. GO (**a**) and KEGG (**b**) functional enrichment analysis of predicted target genes, (**c**) the sequence of *CPT1A* 3′ UTR region and xeno-miR168b, (**d**) transfection efficiency of xeno-miR168b mimics, (**e**) expression level of *CPT1A* in 293T cells after the overexpression of xeno-miR168b, (**f**) dual-luciferase reporter assay for WT-*CPT1A* and MUT-*CPT1A* vectors in HEK-293T cells with or without xeno-miR168b overexpression, (**g**) gene expression level of *AMPK* and *ACC*, (**h**) WB analysis of *AMPK* and *p-AMPK*, and (**i**) quantitative analysis of protein bands in (**h**).

Bioinformatics analysis predicted 1834 potential target genes of xeno-miR168b in dairy cows. The predicted target genes were analyzed for Gene Ontology (GO) and Kyoto Encyclopedia of Genes and Genomes (KEGG) functional enrichment analysis. GO functional enrichment analysis identified significantly enriched ATP binding as a functional characteristic of potential target genes of xeno-miR168b (Figure 8a). In addition, KEGG enrichment analysis uncovered enrichment in the N-glycan biosynthesis, glycerophospholipid metabolism, and NF-κB signaling pathways (Figure 8b) among the potential target genes.

*CPT1A*, a key gene in lipid metabolism, has a xeno-miR168b binding site mapped to the 3′UTR and was identified as one of the high-scoring candidate genes for xeno-miR168b targeting. To determine whether *CPT1A* is regulated by xeno-miR168b, we cloned WT or MUT 3′UTR sequences of *CPT1A* (Figure 8c) and co-transfected this vector with miR-168b mimics or with NC in HEK-293T cells. We confirmed that xeno-miR168b mimics were efficiently expressed in this system (Figure 8d; *p* < 0.01). *CPT1A* expression was significantly decreased in miR-168b-transfected HEK-293T cells compared with the control (*p* < 0.01; Figure 8e). Further, we observed markedly lower luciferase activity in cells transfected with the WT-*CPT1A* vector compared with NC (*p* < 0.01), but no significant difference in relative luciferase activity was observed between cells harboring the MUT-*CPT1A* vector and NC control (Figure 8f). These results demonstrated that *CPT1A* was a target gene of xeno-miR168b.

Finally, the experiment demonstrated that the *AMPK* signaling pathway was activated by xeno-miR168b overexpression, which resulted in suppressed expression of acetyl-CoA carboxylase (*ACC*), which is a downstream target that is inhibited by *AMPK* activity (*p* < 0.01; Figure 8g–i). *AMPK* signaling regulates fatty acid oxidation and *ACC* participates in de novo synthesis of fatty acids; thus, our results suggested that xeno-miR168b regulated *AMPK* signaling and impaired fatty acid oxidation and the de novo synthesis of fatty acids.

### 3.7. CPT1A Silencing Inhibited Lipid Droplet Formation in BMECs

**Figure 9:**
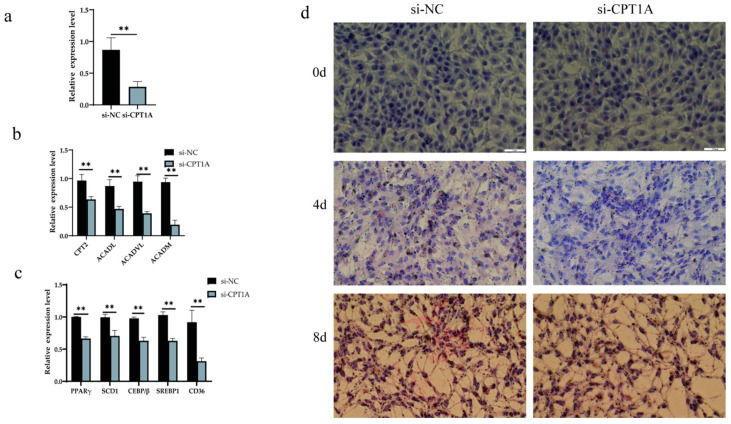
Effect of si*CPT1A* on lipid metabolism in BMECs. Data are given as the mean ± SD for *n* = 3/group. ** *p* < 0.01 were considered statistically significant. (**a**) Gene interference efficiency, (**b**) expression of genes downstream of *CPT1A* in *CPT1A*-silenced cells, (**c**) expression of lipid metabolism genes, and (**d**) lipid droplet formation in BMECs after *CPT1A* silencing.

To reveal the effect of *CPT1A*, which is a target gene of xeno-miR168b, on lipid metabolism in BMECs, we infected BMECs with *CPT1A*-targeting or non-targeting control siRNAs, and we confirmed the efficiency of the knockdown (*p* < 0.01; Figure 9a). Si*CPT1A* robustly decreased the expression of genes downstream of *CPT1A* (*p* < 0.01; Figure 9b). In addition, *CPT1A* silencing significantly decreased the expression levels of *CD36*, *PPARγ*, *SCD1*, *C/EBPβ*, and *SREBP1* (*p* < 0.01), linking *CPT1A* silencing to the inhibition of triglyceride synthesis, brown fat production, milk fat synthesis, and fatty acid absorption (Figure 9c). We further confirmed, via Oil Red O staining, that si*CPT1A* reduced the formation of lipid droplets in BMECs (Figure 9d). The results indicated that xeno-miR168b could inhibit fat production in BMECs by targeting *CPT1A*.

## 4. Discussion

In this study, xeno-miRNAs were detected in the blood and milk of both high-fat-milk and low-fat-milk dairy cows, which demonstrated that xeno-miRNAs could be absorbed into the tissues of dairy cows. This was consistent with previous studies that demonstrated direct uptake of plant miRNAs into animals without special treatment [4,10,27,28,29]. The expression levels of xeno-miRNAs in blood and milk were statistically different in dairy cows within the same group (high-fat group or low-fat group), which might have been related to their variable intake and/or metabolic traits of individual dairy cows. There were also some differences in the milk yield of cows collected in the experiment. An earlier experiment suggested a potential negative correlation between milk yield and milk fat rate, where a higher total milk solids yield was driven by higher fat and protein composition from a reduced milk volume [30]. However, this experiment only collected a fixed volume of milk for RNA extraction and concentration measurement. In the case of a fixed volume, the relative expression level of miRNA is hardly affected by milk production. Therefore, milk production is not listed as the main factor that affected the expression level of miRNA in this study. The final results showed that xeno-miR168b levels were more consistent in the blood and milk of dairy cows within the same cohort, but the levels were significantly different between the high-fat-milk and low-fat-milk dairy cows. Therefore, we speculated that xeno-miR168b may have influenced the activity of genes that control lipid metabolism in dairy cows.

This study demonstrated that the overexpression of xeno-miR168b significantly decreased the expression of several lipid metabolism genes, including *PPARγ*, *SCD1*, *C/EBPβ*, and *SREBP1* genes, compared with the NC group. *PPARγ* is one of the main transcriptional regulators of lipid metabolism in mammals, where it regulates lipid metabolism, glucose metabolism, and adipocyte proliferation and differentiation [31]. *SCD1* is involved in TG synthesis, and *CEBP/β* is the main switch that turns on brown fat gene expression. *SREBP1* is an important factor that regulates the expression of genes related to milk fat synthesis. Thus, our findings implicated the *PPARγ* signaling pathway, TG synthesis, brown fat conversion, and milk fat synthesis as pathways that contributed to the low-milk-fat phenotype associated with high xeno-miR168b expression. The inhibitory effect of alfalfa-derived xeno-miR168b on milk fat synthesis was consistent with that of rice-derived miR-168a on *LDL*s [7]. Moreover, the regulating effect of alfalfa-derived xeno-miR168b is merely one of multiple coordination genes that are involved in a complex regulatory network for milk fat synthesis in dairy cows. Therefore, more work is required to uncover the absorption, transport, metabolism, and integrated effect mechanism of alfalfa exogenous miRNA in cow milk fat.

Bioinformatics analysis determined that alfalfa xeno-miR168b could specifically target *CPT1A*, a subtype of carnitine palmitoyl transferase 1 (*CPT1*), which has been linked to lipid metabolism in dairy cows. Previous studies showed that alfalfa-derived miR-5754 could cross-kingdom regulate metastasis-associated lung adenocarcinoma transcript 1 (*MALAT1*) to promote cell proliferation. This showed that the alfalfa-derived miRNA had the ability to cross-kingdom target animal mRNA to exert regulatory effects [32]. *CPT1A* was shown to regulate fatty acid oxidation [33,34], and many studies showed that *CPT1A* is closely related to fat deposition in animals; this is consistent with our finding that xeno-miR168b affected *CPT1A* expression to regulate fat metabolism in BMECs [35,36].

**Figure 10:**
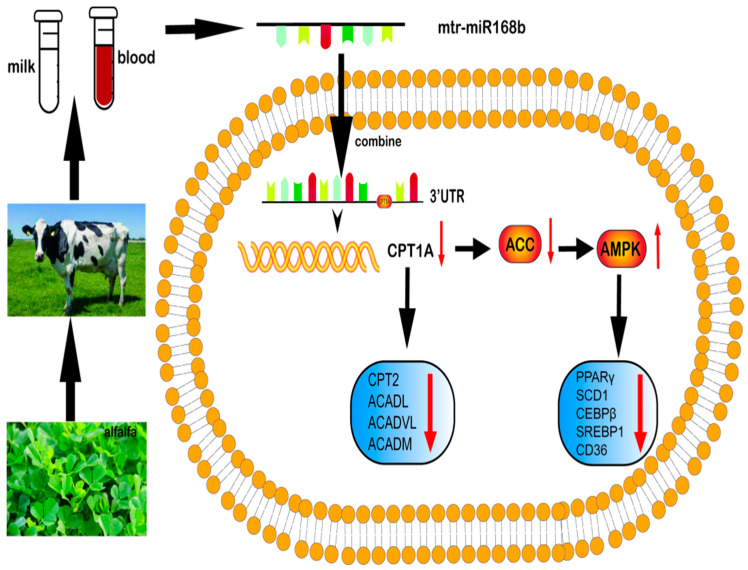
Summary of the pathway by which xeno-miR168b from alfalfa influences lipid metabolism in BMECs.

*AMPK* is the main regulator of energy metabolism in cells and organisms. When intracellular ATP decreases, which results in an increased AMP:ATP ratio, *AMPK* is activated [37]. *ACC* is a rate-limiting enzyme of fatty acid metabolism that plays an important role in fatty acid metabolism and it is inhibited by *AMPK* activity. Previous studies showed that extracts from Chinese herbal medicinal Rehmannia glutinosa and mulberry leaves can improve lipid metabolism disorder in diabetic mice by phosphorylating *AMPK*, demonstrating that plant-derived molecular nutrients can regulate lipid metabolism in animals through *AMPK* signaling [38,39]. Our results suggested a model in which milk fat content in dairy cows can be regulated by xeno-miR168b, which decreases *CPT1A* expression and downregulates *AMPK/ACC* signaling (Figure 10). Decreased *AMPK* activity reduces the synthesis of malonyl-CoA in vivo and activates *CPT1A*, which promotes the oxidation of long-chain fatty acyl-coenzyme A from the cytoplasm into the mitochondria. This would reduce the deposition of lipids in peripheral tissues and enhance the oxidation of fatty acids, resulting in a decrease in TG content and lipid droplet formation, which we observed in BMECs after the overexpression of xeno-miR168b [40].

In order to further clarify the effect of mtr-miR168b-targeted inhibition of *CPT1A* expression on BMECs lipid metabolism, we knocked down the gene expression level of *CPT1A*. Quantitative detection showed that the expression levels of downstream genes and lipid metabolism-related genes of *CPT1A* knockdown pathway were significantly reduced. *PPAR* (including *PPARγ*) is generally considered an upstream regulator of the *CPT1A* gene. However, not all signal pathways involved in *CPT1A* are directly involved in *PPAR*, such as the fatty acid β oxidation (Bostaurus) signaling pathway. Therefore, the silence effect of *CPT1A* cannot be affected by *PPARγ* silencing. Xeno-miR168b can only regulate *PPAR* or other signal-pathway-related genes by regulating target genes, but not *PPAR* directly. At the same time, we believe that high expression of *CPT1A* can improve *PPAR* expression and save lipid metabolism. Long-chain acyl-CoA dehydrogenase (*ACADL*), very long-chain acyl-CoA dehydrogenase (*ACADVL*), and medium-chain acyl-CoA dehydrogenase (*ACADM*) are all acyl-CoA dehydrogenases, which are important coenzyme families involved in fatty acid metabolism. *ACADL* plays an important role in the β oxidation of long-chain fatty acids, and its deficiency or deletion will cause mitochondrial dysfunction and then affect lipid metabolism [41]. *ACADVL* and *ACADM* genes can participate in biological processes related to lipid metabolism, affect the synthesis and decomposition of fatty acids and glycerol, and play a vital role in lipid metabolism [42,43,44]. The low expression of *CPT1A* affects the fatty acid metabolism of BMECs and inhibits the synthesis and accumulation of cellular lipids, which is also consistent with the result that the overexpression of xeno-miR168b inhibits fatty acid production.

Milk fat is one of the main indicators used to measure the quality of milk. For calves, milk fat also plays a very important role. When calves cannot get enough energy from milk, fat supplements need to be added to the feed to meet the low energy density per unit of feed for calves. However, fat supplementation has adverse effects on the rumen fermentation of young cattle [45,46,47]. A. Karimi et al. showed that a treatment group of newborn Holstein female calves that had their feed supplemented with soybean oil (SBO) had a reduced starter intake, average daily gain (tendency), and fecal consistency compared with unsupplemented diets. In addition, the negative effects of SBO supplementation were exacerbated when alfalfa hay (AH) was incorporated into the starter feed [48]. Recent studies showed that the effect of adding CS (corn silage) and PF (rumen-inert palm fat acids) to a calf diet is better than that of AH and SBO [49]. The above results may be related to the regulation of lipids from nutrients in alfalfa. Alfalfa contains a large amount of dietary fiber, which helps to reduce the cholesterol level in animals [50,51]. In addition, saponin, which is one of the main components of secondary metabolites in alfalfa, can also reduce the cholesterol level and *LDL* and inhibit the activity of cholesterol-related synthase [52,53]. L. Zhang et al. detected plant miRNAs in calves and verified that plant miR168 could target *LDLRAP1* in the livers of mice and humans, which slows the clearance of blood *LDL*s [7]. The above results indicate that alfalfa and plant-derived miR168 have inhibitory effects on lipid metabolism, which is consistent with the results of this study.

## 5. Conclusions

This study revealed that alfalfa xeno-miR168b can be detected in the milk and blood of dairy cows, and the expression of alfalfa xeno-miR168b was significantly higher in cows that produced low-fat milk compared with cows that produced high-fat milk. The overexpression of xeno-miR168b in BMECs inhibited proliferation, promoted apoptosis, and reduced lipid droplet formation and TG accumulation. Genetic analysis indicated that these effects were executed via the xeno-miR168b-mediated regulation of *CPT1A* and the *AMPK/ACC* signaling pathway. These findings uncovered a novel regulatory pathway of dairy cow milk fat production that has important implications for dietary strategies to improve the nutritional content of milk from ruminant animals.

## Figures and Tables

**Table 1:** Sampling cattle basic information.

ID	Calving Date	Parity	Calving Interval (days)	Lactation Days
0001	19 July 2021	4	371	120
0002	12 July 2021	4	340	127
0003	13 May 2021	4	364	187
0004	12 July 2021	4	426	127
0005	15 May 2021	4	381	185
0006	31 July 2021	4	364	108

**Table 2:** The dietary composition and nutrient level (DM basis, %).

Item	Content	Nutrient Level	Content
Alfalfa	6.00	Milk net energy production (NEL)/(MJ/kg^−1^)	7.78
Corn silage	50.00	Crude protein (CP)	17.2
Cottonseed	1.00	Neutral detergent fiber (NDF)	27.5
Beet granules	1.60	Fat	3.9
Wet beer lees	10.00	Acid detergent fiber (ADF)	21.7
Tablet corn	4.00	Ca	0.53
5% premix	26.96	P	0.36

The premix contained the following per kilogram: VA 800,000 IU, VD 200,000 IU, VE 4000 mg, Cu 1200 mg, Fe 6000 mg, Zn 4000 mg, Co 40 mg, and Se 32 mg.

**Table 3:** Primer sequences of miRNA.

miRNA Name	Primer Sequence (5′-3′)
U6	F: GCTTCGGCAGCACATATACTAAAAT R: CGCTTCACGAATTTGCGTGTCAT
mtr-miR168b	F: CATGTGTCGCTTGGTGCAG R: AGTGCAGGGTCCGAGGTATT RT: GTCGTATCCAGTGCAGGGTCCGAGGTATTCGCACT GGATACGACTTCCCGAC
mtr-miR166a	F: CACAGTTCGGACCAGGCTT R: AGTGCAGGGTCCGAGGTATT RT: GTCGTATCCAGTGCAGGGTCCGAGGTATTCGCACT GGATACGACGGGGAATG
mtr-miR168c-3p	F: CATAGACCCGCCTTGCATC R: AGTGCAGGGTCCGAGGTATT RT: GTCGTATCCAGTGCAGGGTCCGAGGTATTCGCACTG GATACGACATTCAGTT
mtr-miR156f	F: CCGTTGACAGAAGATAGAGAGCAC R: ATCCAGTGCAGGGTCCGAGG RT: GTCGTATCCAGTGCAGGGTCCGAGGTATTCGCACT GGATACGACGTGCTC
mtr-novel-miR54	F: CCAAGTCCTTGTGTTGCATCTC R: ATCCAGTGCAGGGTCCGAGG RT: GTCGTATCCAGTGCAGGGTCCGAGGTATTCGCACT GGATACGACGAGATG
bta-miR-16a	F: GCCCGTAGCAGCACGTAAAT R: TGTCGTGGAGTCGGCAAT RT: CTCAACTGGTGTCGTGGAGTCGGCAATTCAGTTG AGTCAGAC

F: forward primer; R: reverse primer; RT: reverse transcription primer.

**Table 4:** Primer sequences of the genes.

Gene	Primer Sequence (5′-3′)
*GAPDH*	F: GGCATCGTGGAGGGACTTATG R: GCCAGTGAGCTTCCCGTTGAG
*CDK4*	F: GTGACAAGTGGTGGGACAGT R: GATACAGCCAACGCTCCACA
*Cyclin D1*	F: CATGAACTACCTGGACCGCT R: TCTTGGAGAGGAAGTGCTCG
*Cyclin D2*	F: CACCGATGTGGATTGCCTCA R: TCCAGCTCATCCTCCGACTT
*PCNA*	F: GAACCTCACCAGCATGTCCA R: ACGTGTCCGCGTTATCTTCA
*BAX*	F: GAGATGAATTGGACAGTAACA R: TTGAAGTTGCCGTCAGAA
*PPARγ*	F: AAAGGAGAGCCTGAACTTGGAG R: TCTGAACTGTGCTGTGGCAA
*SCD1*	F: ACATTGATCCCCACCTGCAA R: AAACGTCATTCTGGAACGGC
*CEBP/β*	F: TGGTGAATAGTGCTGCCCAT R: GGTGGTAGTTGTGGAAGCCC
*SREBP1*	F: CAATGTGTGAGAAGGCCAGT R: ACAAGGAGCAGGTCACACAG
*CD36*	F: TCCTGGACCCTGAACACT R: ATAATGCCTTGCTGATGC
*CPT2*	F: CCTTCCTTCCTGTCTTGGTATG R: TTCAGAGGCACTCACAATGTTC
*ACADM*	F: CCAAAGACAGAAAAGAAG R: TATACAACAGACCAAAGG
*ACADVL*	F: TACCCTCAACGGAAGCAA R: TGTTGGCACTCACCATGTAC
*ACADL*	F: AGGGAAATGTATTGGTGC R: CTGGCTGAAACTGCTATCT
*CPT1A*	F: TTGCGGCCGC CTTAAGGGACAAGCGATT R: CCTCGAG CAGTCTGATGGAAGGGAA
*AMPK*	F: TCTGCCGTGGATTACTGT R: AGCCTGCCTGAGATGACT
*ACC*	F: CCACGGAACCTTGACTACGA R: CATCAGCGACAGATGCGATA

**Table 5:** miRNA sequences and TPM in alfalfa.

miRNA	Sequence (5′-3′)	TPM in ZM1	TPM in XY52
mtr-miR5743a	TGAGAACTGTTTTCCGCACCTT	6019.36	61.23
mtr-miR5743b	TGAGAACTGTTTTCCGCACCTT	6019.36	61.23
novel_miR_158	AAAGGAUCAUUGGAUAAGUUC	1034.19	57.55
mtr-miR5754	TATTGCACTCATCTTCCATGGC	904.99	6.80
novel_miR_54	AAGUCCUUGUGUUGCAUCUC	645.52	1877.09
mtr-miR156f	TTGACAGAAGATAGAGAGCAC	163.666	10.66
mtr-miR156e	TTGACAGAAGATAGAGAGCAC	163.66	10.66
mtr-miR156h-5p	TTGACAGAAGATAGAGAGCAC	163.66	10.66

**Table 6:** DHI analysis of milk (%).

ID	Item	Milk Production/kg	Fat Percentage	Protein Percentage	Lactose Percentage	Total Solids
0001	High-milk fat group	44.7	4.22	3.27	5.78	14.62
0002		43.8	4.21	3.24	4.32	13.44
0003		34.9	4.27	3.26	5.21	15.21
0004	Low-milk fat group	33.0	3.42	3.1	5.38	13.32
0005		31.2	3.50	3.09	5.59	13.88
0006		29.8	3.58	3.21	4.79	13.19

## Data Availability

The raw data of the alfalfa miRNA sequencing library can be accessed with the following link: http://www.ncbi.nlm.nih.gov/bioproject/822492, accessed on 27 July 2022.
